# Mitochondrial dysfunction in *Trypanosoma cruzi*: the role of *Serratia marcescens *prodigiosin in the alternative treatment of Chagas disease

**DOI:** 10.1186/1756-3305-4-66

**Published:** 2011-05-06

**Authors:** Carlos Genes, Eduard Baquero, Fernando Echeverri, Juan D Maya, Omar Triana

**Affiliations:** 1 Grupo Biología y Control de Enfermedades Infecciosas BCEI - SIU, Instituto de Biología, Universidad de Antioquia, Medellín, Colombia; 2 Grupo de Química de Productos Naturales - SIU, Instituto de Biología, Universidad de Antioquia, Medellín, Colombia; 3 Programa de Farmacología Molecular y Clínica. ICBM. Facultad de Medicina. Universidad de Chile, Santiago, Chile

## Abstract

**Background:**

Chagas disease is a health threat for many people, mostly those living in Latin America. One of the most important problems in treatment is the limitation of existing drugs. Prodigiosin, produced by *Serratia marcescens (Rhodnius prolixus *endosymbiont), belongs to the red-pigmented bacterial prodiginine family, which displays numerous biological activities, including antibacterial, antifungal, antiprotozoal, antimalarial, immunosuppressive, and anticancer properties. Here we describe its effects on *Trypanosoma cruzi *mitochondria belonging to Tc I and Tc II.

**Results:**

Parasites exposed to prodigiosin altered the mitochondrial function and oxidative phosphorylation could not have a normal course, probably by inhibition of complex III. Prodigiosin did not produce cytotoxic effects in lymphocytes and Vero cells and has better effects than benznidazole. Our data suggest that the action of prodigiosin on the parasites is mediated by mitochondrial structural and functional disruptions that could lead the parasites to an apoptotic-like cell death process.

**Conclusions:**

Here, we propose a potentially useful trypanocidal agent derived from knowledge of an important aspect of the natural life cycle of the parasite: the vector-parasite interaction. Our results indicate that prodigiosin could be a good candidate for the treatment of Chagas disease.

## Background

Chagas disease continues to represent a health threat for an estimated 28 million people, most of them living in Latin America. One of the most important problems in the outcome of Chagas disease is the limitation of existing drugs for treatment [1]. For more than 40 years, only two drugs, nifurtimox and benznidazole, have been available to treat Chagas disease. Both have limited efficacy (about 80% efficacy in the acute phase and lower in the chronic phase), as well as frequent and significant side effects [2]. Other potentially beneficial drugs, such as allopurinol or itraconazole, do not have a high enough degree of clinical efficacy, as compared with nifurtimox or benznidazole; Posaconazole is a promising drug, but expensive [2]. Furthermore, hundreds of natural and synthetic compounds have been tested against the protozoan parasite *Trypanosoma cruzi*, the causative agent of Chagas disease. However, very few are devoid of cytotoxic activity or have proved more efficacious than nifurtimox and benznidazole, especially against the intracellular amastigotes [3]. Therefore, disease control is mainly based on the elimination of insect vectors. Most species of the Triatominae (Hemiptera-Reduviidae) subfamily are potential vectors of *T. cruzi*. It has been reported that in the first few days after a blood meal, the number of bacteria in the anterior midgut (stomach) of *Rhodnius prolixus *increases dramatically. In addition, many of the bloodstream trypomastigotes are lysed in the vector's stomach. This probably occurs as part of a complex ecological interaction in the vector, where bacteria play a central role [4]. A wide variety of bacteria including species from the *Actinobacteria*, *Firmicutes*, and *Proteobacteria *have been detected in the triatomine midgut tract [5,6]. It has been reported that *Serratia marcescens *biotype A1a has trypanolytic activity in the gut of the vector *Rhodnius prolixus*, especially on the Y strain [7]. The prodigiosin produced by this bacterium could be responsible for the trypanocidal activity observed in the vector.

Prodigiosins, which belong to the red-pigmented bacterial prodiginine family, are tripyrrolic compounds that display numerous biological activities, including antibacterial, antifungal, antiprotozoal, antimalarial, immunosuppressive, and anticancer properties [8,9]. This broad spectrum of activities might be related to their capacity to alter key proteins involved in cell cycle or intracellular signal transduction or to induce apoptosis [10]. Certainly, there is abundant evidence indicating that bacterial prodiginines and synthetic derivatives are effective proapoptotic agents with multiple cellular targets, and they are active against numerous cancer cell lines, including multidrug-resistant cells [9]. Very important is the demonstration of little or no toxicity toward normal cell lines. For this reason a synthetic derivative of prodiginines, GX15-070 (Obatoclax), developed through structure-activity relationship studies of the pyrrolic ring A of GX15, is in multiple Phase I and II clinical trials in both single- and dual-agent studies to treat different types of cancer [8,11].

Therefore, prodiginines have real therapeutic potential and although their mode of action is not yet clear and can vary depending on cell type, it has been reported that their effects can strongly compromise the mitochondrial metabolism. The mitochondrial disturbances can be evidenced by alterations in cellular respiration, energy balance, or even structural modifications on the organelle as proposed recently [12-14]. All these alterations suggest that the prodigiosin effects could result in an apoptotic process on cells [13-15].

Here we provide data on the selective trypanocidal activity of prodigiosin, evidenced by low cytotoxicity against a normal cell line and human lymphocytes. Additionally, we compare the effects of several mitochondrial electron transport chain (ETC) inhibitors, to propose the mitochondria as a possible target for prodigiosin effects on *T. cruzi*.

## Results

### Trypanocidal activity of prodigiosin and electron transport chain inhibitors

**Figure 1:**
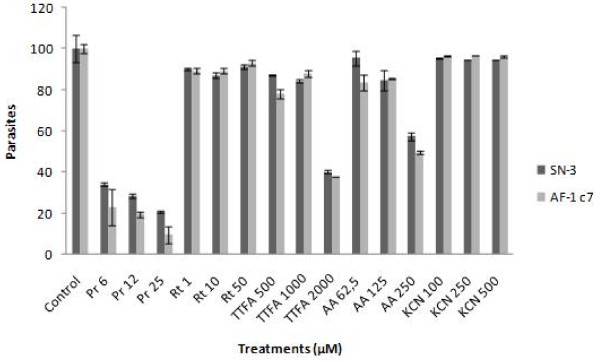
**Activity of prodigiosin against *Trypanosoma cruzi *epimastigotes of the SN-3 strain and AF1 clone 7**. 2 × 10^6^epimastigotes were treated with different concentrations of prodigiosin for 24 h and its viability was evaluated by flow cytometry using propidium iodide.

**Figure 2:**
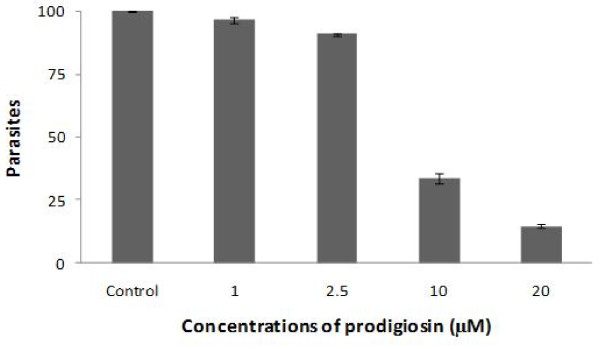
**Activity of prodigiosin against *Trypanosoma cruzi *trypomastigotes of the SN-3 strain**. 2 × 10^6^parasites were treated with different concentrations of prodigiosin for 24 h and its viability was evaluated by flow cytometry using propidium iodide.

Axenic cultures of *T. cruzi *epimastigotes were treated with different concentrations of rotenone, TTFA, KCN, antimycin A, or prodigiosin for 24 h. Rotenone and KCN had no effect on *T. cruzi *viability, while TTFA and antimycin A decreased the viability by 60 and 50%, respectively. Both parasite strains tested (SN-3 and AF-1c7) had similar responses to mitochondrial inhibitors (Figure 1). The activity of prodigiosin showed higher trypanocidal activity than the other mitochondrial inhibitors assayed. SN-3 and AF-1 c7 parasites showed a small differential susceptibility to prodigiosin treatment. SN-3 IC_50 _was 2.7 μM while AF1 c7 IC_50 _was 2.2 μM (p < 0.05) (Figure 1). The IC_50 _to benznidazole was 34.62 and 4.69 to SN-3 and AF1. SN-3 trypomastigotes treated with prodigiosin presented an IC_50 _of 3.2 μM, which is slightly higher than epimastigote IC_50 _(Figure 2). The *T. cruzi *AF-1 clone was unable to infect Vero cells. Thus, the prodigiosin effect on trypomastigote viability against this clone was not determined.

### Cytotoxicity assay

**Figure 3:**
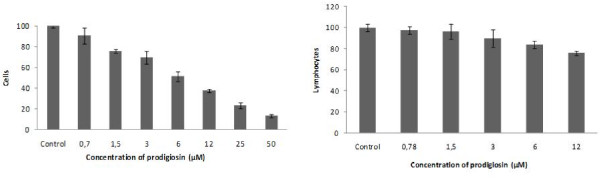
**Activity of prodigiosin against Vero cells and human lymphocytes**. 2.5 × 10^6 ^adherent Vero cells were treated with prodigiosin for 24 h. After incubation, the supernatant and the adherent cells, previously dislodged, were used to quantify live cells by propidium iodide. 2 × 10^5 ^lymphocytes were treated with prodigiosin for 24 h. The lives cells were counting by optical microscopy using the trypan blue dye.

To evaluate prodigiosin selectivity, Vero cells and human lymphocytes were treated with different concentrations of the compound for 24 h. Indeed, as observed in Figure 3, prodigiosin had a slightly toxic effect on the viability of Vero cells, with IC_50 _6.49 ± 0.8 and a selectivity index (IC_50 _Vero cells/IC_50 _parasites) of 2.2 (SN3) and 2.49 (AF1 c7). However, on human lymphocytes, the toxic effects of prodigiosin were minimal with less of 25% cell death at 6 times the IC_50 _concentration (Figure 3).

### Mitochondrial function assays

#### Effects on oxygen uptake

**Figure 4:**
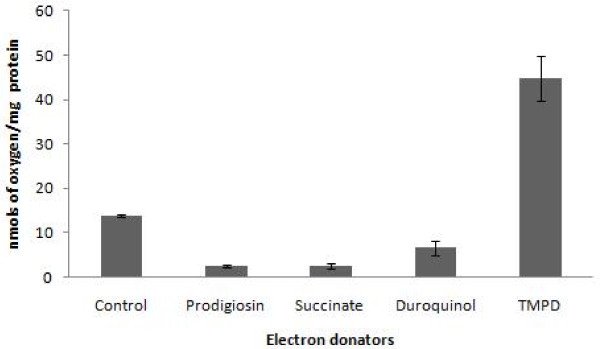
**Effects of prodigiosin on mitochondrial electron transport chain**. Electron donators were added after the addition of prodigiosin (0.287 μM to 5 mM) to the oxygen consumption reaction mix, and oxygen uptake was determined.

To study the prodigiosin effects on oxygen uptake, we used the mitochondrial inhibitors TTFA and KCN as controls. Table 1 shows the effects of TTFA, KCN, and prodigiosin on oxygen uptake. KCN inhibited mitochondrial respiration by 50%, while prodigiosin and TTFA inhibited parasite respiration by more than 80%. These results indicate that the mitochondrial function is altered and that oxidative phosphorylation does not take a normal course. On the other hand, the addition of duroquinol, but not succinate, to prodigiosin-treated epimastigotes re-established and increased oxygen uptake, indicating that inhibition of respiration in *T. cruzi *could occur mainly due to the blockade of complex III (Figure 4). No effect of DMSO alone, in which prodigiosin was dissolved, was observed.

**Table 1:** Effect of prodigiosin, TTFA, and KCN on mitochondrial respiration.

	Oxygen uptake	Oxygen uptake after succinate	Oxygen uptake after duroquinol	Oxygen uptake after TMPD/ascorbate
	**nmol O**_**2 **_**/min/mg protein**	**nmol O**_**2 **_**/min/mg protein**	**nmol O**_**2 **_**/min/mg protein**	**nmol O**_**2 **_**/min/mg protein**

**Control**	13.88889	___	___	___

**Prodigiosin (5 mM)**	2.46667	2.46667	6.53333	44.66667

**TTFA (5 mM)**	2.23333	ND	ND	ND

**KCN (5 mM)**	6.76722	ND	ND	ND

Oxygen consumption was evaluated in control and treated parasites. In order to determine which complexes of the electron transport chain were involved in the abnormal respiration, we added electron donors for each one of the complexes. The untreated parasites showed a higher oxygen uptake with respect to prodigiosin TTFA- and KCN-treated parasites. However, when electron donors for complex II and III were added the oxygen uptake was increased.

### Mitochondrial transmembrane potential (Ψm) measurements

**Figure 5:**
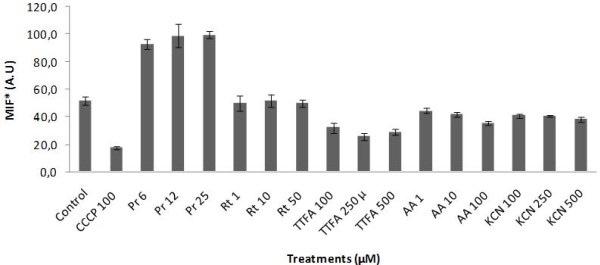
**Effect of prodigiosin on mitochondrial transmembrane potential of epimastigotes of *T. cruzi *SN-3 strain**. 1.5 × 10^6 ^parasites/mL were treated with rotenone, TTFA, antimycin A, KCN, or prodigiosin for 1 h. The parasites were then incubated with TMRM and analyzed with a cytometer. The uncoupler CCCP (100 μM) was used as positive control. *MIF: Medium intensity of fluorescence.

We studied the Ψm through incorporation of the TMRM probe, to determine whether the effects of prodigiosin on *T. cruzi *mitochondria were associated with the metabolic function of this organelle. We characterized the *T. cruzi *Ψm using the classic mitochondrial inhibitors rotenone, TTFA, KCN, and antimycin A. Subsequently, parasites were incubated with TMRM after 1 h of treatment with prodigiosin or mitochondrial inhibitors, and TMRM fluorescence was measured by flow cytometry after 30 min of incubation. The uncoupler CCCP was used as positive control. Our results show that both rotenone and KCN have little effect on Ψm, while TTFA and antimycin A decrease the Ψm after 1 h of treatment (Figure 5). Conversely, prodigiosin demonstrated a dose-dependent membrane hyperpolarization. This hyperpolarization was similar in both the *T. cruzi *SN-3 strain and AF-1 c 7 (data not shown), indicating that the mechanism of action in both parasite strains is similar.

## Discussion

Herein we report the activity of prodigiosin against *T. cruzi*, and provide evidence that its mode of action involves the mitochondrial function. Current therapy for Chagas disease is not 100% effective. This picture is complicated by the fact that nifurtimox and benznidazole produce a wide variety of adverse events that may necessitate suspension of treatment. In addition, several strains have been identified that are resistant to these drugs [1].

Diverse approaches have been used to search for new trypanocidal compounds, from natural and synthetic sources. Here, we propose a potentially useful trypanocidal agent that is derived from knowledge of an important aspect of the natural life cycle of the parasite: the vector-parasite interaction. Indeed, basic biological factors such as the food supply, intestinal components, gut flora, and insect physiology, which are relevant to understanding the parasite-vector interaction reveal new perspectives for the control of Chagas disease. Although *Trypanosoma cruzi *and triatomines probably did not co-evolve to facilitate protozoan transmission [16], it is clear that Chagas disease is dependent on a high degree of interaction between the triatomine vectors and the parasites.

The co-evolution of parasites and insects necessarily promoted the development of strategies based on both insect vector and parasite mechanisms, maintaining an ecological equilibrium that might facilitate parasite development in the invertebrate host. Multiple factors are present in the insect vector gut that explain the establishment of *T. cruzi *colonization. One outstanding factor is the potential influence of the gut microbiota on the parasite life cycle. In this respect, following a blood meal, bacterial populations in the triatomines' midgut often undergo massive rapid expansions, as much as 10,000-fold [7]. Many microbial factors may be produced in the vector gut to target parasites ingested with a blood meal. These include pigments such as prodigiosin that are produced by the Gram-negative bacteria *S. marcescens*, *S. plymuthica*, and Streptomyces [17], all of which occur in the midgut of vector insects. Prodigiosin induces apoptotic phenomena and cell death in cancer cell lines [18]. In addition, it has been reported to have a strong inhibitory effect on a V79 fibroblast cell line [19]. However, other studies failed to demonstrate cytotoxicity against normal cells as compared with tumor cells lines [20]. In addition, prodigiosin derivatives show marked activity against *Plasmodium falciparum *[21], and strong activity against *T. cruzi *has also been reported [19].

To study the mode of action of prodigiosin on *T. cruzi*, we focused our research on the mitochondrial function. *T. cruzi *has only one mitochondrium, which is fundamental to parasite survival and regulation of the processes related to energy balance and apoptosis modulation. We focused on two points in mitochondrial metabolism. The first was oxygen consumption, a key step in oxidative phosphorylation inside the mitochondrial matrix, and the second was the mitochondrial membrane potential **(Ψm)**. Ψm is crucial for ATP production by ATP synthase, and it is also involved in protein transport inside the organelle and in regulation of the glycolysis process. Moreover, its variations are used as an earlier marker of mammalian and protozoan programmed cell death (PCD) progression.

In order to validate our experimental strategy, we studied the effects of classic mitochondrial inhibitors. The results showed that rotenone and KCN, electron transport chain inhibitors of complex I and IV, respectively, do not have an effect on *T. cruzi *viability, and caused slight or no Ψm and cellular respiration alterations. Our data confirm the results obtained by other groups suggesting that *T. cruzi *complex I is absent or at least cannot be inhibited by rotenone [22] and complex IV might have an alternative pathway, probably mediated by a trypanosome alternative oxidase as described for *T. brucei *[23,24]. The existence of mitochondrial complex I in trypanosomatids is still a matter of intense discussion. Recently, a very detailed bioinformatic analysis to predict the composition of a putative trypanosomatid complex I revealed the presence of all subunits known to be involved in complex I-electron transport, but four membrane subunits assumed to be involved in proton extrusion are missing, suggesting that this complex is not involved in energy transduction [25]. In this sense, experimental data obtained in *T. cruzi natural *strains containing deletions in the ND4, ND5 and ND7 genes coding for complex I subunits demonstrated that this complex is not functional in NADH oxidation and also is not involved in energy transduction [26]. Furthermore, a RNAi study against the putative subunits of complex I of *T. brucei *revealed the presence of this complex and its participation in electron transport, although this complex does not play an important role in energy metabolism of the *T. brucei *procyclics [27]. Taking together, these results and those reported in this study, indicate that the function of mitochondrial complex I remain a very intriguing issue to be studied.

On the other hand, the treatment with TTFA, a succinate dehydrogenase (complex II) inhibitor, was able to induce considerable dysfunction of the *T. cruzi *mitochondria, which was evidenced by a decrease in membrane depolarization, respiration and cell viability. It has been suggested that the complex II in *T. cruzi *is the main electron sink from the Krebs cycle and its importance in energy transduction is clear [22]. Similarly, the treatment with the complex III inhibitor antimycin A showed a similar effect to the TTFA, inducing a loss of mitochondrial intermembrane potential and decreasing respiration and cell viability. All of these results are in concordance with previous reports showing the importance of complex II and III in the maintenance of mitochondrial stability in *T. cruzi*.

On the other hand, potassium cyanide was able to induce the blockage of cellular respiration in at least 50% of oxygen uptake. Similar results have been reported in other mitochondrial respiration studies [28,29]. KCN treatment did not induce a decrease on the Ψm or even on parasite viability. It has been suggested that *T. cruzi *have three respiratory terminals: 1) A respiratory terminal sensitive to cyanide and azide; 2) a cyanide-sensitive but azide-insensitive terminal; and 3) a terminal insensitive to both inhibitors.

Taking all the results obtained with the mitochondrial electron transport chain inhibitors, we concluded that the behavior and measurements made show the methodology's confidence in studying other different stimuli such as prodigiosin.

The mitochondrial alterations observed during prodigiosin treatment included an important inhibition of oxygen consumption, probably due to the action of prodigiosin throughout the electron transport chain. The use of electron donators allowed us to propose that one of the effects of prodigiosin inside *T. cruzi *mitochondria could be associated to complex III blockage, although complex II also presented a slight reactivation after succinate addition. To confirm our hypothesis we studied the Ψm behavior. The Ψm is one of the most important events inside the mitochondria of several cell types and usually a decrease of Ψm is associated with PCD [30]. Furthermore, hyperpolarization has been identified as an early event related with H_2_O_2 _[31], p53 [32], and staurosporin-induced apoptosis [33]. Due to Ψm hyperpolarization and extrusion of H ^+ ^ions from the mitochondrial matrix, the cytochromes within the electron transport chain become more reduced, which favors the generation of reactive oxygen intermediates (ROI). Thus, mitochondrial hyperpolarization is a likely cause of ROI production at early stages, representing a key checkpoint in cell-fate decision [34,35]. Indeed, prodigiosin induced a marked hyperpolarization of the mitochondria after 1 h of treatment, suggesting that prodigiosin could act on an early stage of an apoptotic-like cell death pathway. All these results indicate that processes related to the functional and structural stability of mitochondria in *T. cruzi *are altered and could govern the success of programmed cell death pathways in prodigiosin-treated *T. cruzi *parasites.

These findings are consistent with several reports where the mode of action of prodigiosin is related to mitochondrial alterations. The main effects include modifications of oxygen uptake, ATP production, and Ψm that conduce to cell death by an apoptotic process [13]. Recently, an apoptotic cell death process was discovered in protozoan parasites such as *T. brucei*, *Leishmania sp*, and *T. cruzi *[36-38]. In these organisms, the pathways associated to apoptosis progression remain to be elucidated, and the mitochondrial role is unclear; however, some recent works suggest that disturbances of this organelle can induce a similar mammalian apoptotic phenotype [39-45].

Thus, we propose that the anti-*T. cruzi *prodigiosin effect could be facilitated through an apoptotic-like phenomenon mediated by induction of mitochondrial dysfunction, analogous to that reported for cancer cells [13-15]. It is possible to speculate that the apoptotic progression of epimastigotes inside the insect gut might result in at least three different populations of virulent feces (epimastigotes, trypomastigotes, and dead parasites). If the parasitic apoptotic population from the vector is capable of interacting with immune cells of the vertebrate host, it is likely that the resulting attenuation of the immune response might contribute to *T. cruzi *evasion of the host immune response [46]. This would thus facilitate Chagas disease progression [47], as previously demonstrated and suggested in *Leishmania infantum *by Zandbergen *et al*. [48,49].

Finally, our results indicate that prodigiosin could be a good candidate for the treatment of Chagas disease, because we compared the activity of prodigiosin and benznidazole, demonstrating a lower IC_50_. Future research is needed to clarify these findings and the role of prodigiosin in mitochondrial complex III.

## Conclusions

Our results indicate that prodigiosin could be a good candidate for the treatment of Chagas disease. We compared the activity of prodigiosin in two different lineages of *T. cruzi *and both showed a low IC_50_, which is lower than that of benznidazole. However, further research is needed to address these issues and to confirm the advantages of mitochondrial intermembrane potential measurements as a crucial and useful indicator of the biochemical status of kinetoplastid parasites.

## Methods

### Parasites

*Trypanosoma cruzi *epimastigotes of the SN-3 strain (TC I) and AF-1 clone 7 (TC II), both obtained from triatomines, were cultured at 28°C in LIT medium supplemented with 10% FBS. Trypomastigote forms were obtained from SN-3 strain-infected Vero cells and collected from the culture media by centrifugation at 500 *g *for 3 min. The supernatant was discarded and fresh RPMI medium was added to the resultant pellet. Tubes were incubated for 2 h to allow the trypomastigotes to swim out of the pellet. The resulting suspension was then centrifuged and the pellet was suspended in fetal bovine serum-RPMI culture medium at a final density of 2 × 10^7 ^trypomastigotes/mL.

### Bacterial strain

Wild-type *Serratia marcescens *that produces prodigiosin was donated from the School of Medicine, University of Antioquia. *S. marcescens *was characterized using the biochemical test API-20. The bacterial cultures were maintained in nutritive agar for 24 h at 30°C.

### Reagents and inhibitors

The electron transport chain inhibitors Rotenone (specific complex I inhibitor), 2-thenoyltrifluoroactone -TTFA- (specific complex II inhibitor), antimycin A (specific complex III inhibitor), potassium cyanide -KCN- (specific complex IV inhibitor), the uncoupler carbonyl cyanide 3-*cholo*phenylhydrazone (CCCP), and all other reagents used were from Sigma-Aldrich.

### Prodigiosin isolation

Prodigiosin was extracted by *S. marcescens *lysis with a mixture of methanol/1N HCl (24:1). After centrifugation at 2000 g for 10 min, the solvent in supernatant was removed using a rotary evaporator system. Atmospheric pressure liquid chromatography of the extract was performed on a silica gel with dichloromethane and methanol as solvents. The eluted pigmented fractions were pooled and the dichloromethane/methanol extract was vacuum evaporated, redissolved in methanol, and characterized by H-NMR.

### Prodigiosin and inhibitors activity

Epimastigotes and trypomastigotes at a density of 2 × 10^6 ^per mL were treated with different concentrations of mitochondrial electron transport chain inhibitors or prodigiosin for 24 h. Moreover, epimastigotes were exposed to benznidazole with the aim of identifying the IC_50_. Culture growth was followed by flow cytometry with propidium iodide as a viability marker. IC_50 _values were calculated by linear regression analysis of the relation between drug concentration and cell viability.

### Cytotoxicity assay

The effect of prodigiosin on Vero cells and human lymphocytes was evaluated through flow cytometry and microscopy counting, respectively. First, 2.5 × 10^6 ^cells were incubated in RPMI 1640, 2% FCS supplemented medium for 24 h at 37°C with 5% CO_2 _to secure their adherence. Cells were then treated with prodigiosin concentrations ranging from 0.625 μM to 50 μM for 24 h. After treatment, the supernatant and the dislodged adherent cells were used to quantify the live cells by propidium iodide staining. Cells treated with PBS or DMSO 30% were used as negative and positive controls, respectively. The cytotoxic assays for human lymphocytes were carried out using isolated lymphocytes by Ficoll-Paque gradient from a volunteer [50]. A total of 2 × 10^5 ^cells were cultivated in RPMI 1640, 10% FCS supplemented medium for 48 h at 37°C with 5% CO_2_. Lymphocytes were treated with prodigiosin concentrations ranging from 0.78 μM to 12 μM for 24 h. After treatment, the lymphocytes were used to quantify the live cells by trypan blue staining. Lymphocytes treated with PBS or H_2_O_2 _50 μM were used as negative and positive controls, respectively.

### Mitochondrial function assays

#### Oxygen uptake assay

*T. cruzi *epimastigotes were harvested by 500 × *g *centrifugation, washed twice, and suspended in 0.05 M sodium phosphate and 0.107 M sodium chloride buffer, pH 7.4. Respiration measurements were carried out polarographically with a Clark no. 5331 electrode (Yellow Springs Instruments) in a 53 YSI model (Simpson Electric Co) [51]. The chamber volume was 0.6 mL and the temperature was 28°C. The amount of parasite used was equivalent to 1 mg/mL of protein. To study the performance of cellular respiration we used several concentrations of TTFA, KCN, or prodigiosin as treatments. In addition, to study the effect of prodigiosin on succinate dehydrogenase (complex II), cytochrome c reductase (complex III) and cytochrome c oxidase (complex IV), we added succinate 5 mM, duroquinol 0.5 mM, and TMPD 0.1 mM/ascorbate 10 mM, respectively, after having added prodigiosin at concentrations from 0.287 μM to 5 mM. Oxygen uptake was then determined. Values are expressed as mean ± SD for three independent experiments.

### Mitochondrial transmembrane potential (Ψm)

Parasites in LIT medium/10% FCS at 1.5 × 10^6 ^parasites/mL were treated with rotenone, TTFA, antimycin A, KCN, or prodigiosin for 1 h. The treated parasites were then incubated with 1uM tetramethylrhodamine methyl ester perchlorate (TMRM, Sigma CAT# T5428) and analyzed with a cytometer. TMRM loading was carried out at 28°C in all treated and control parasites. The uncoupler CCCP (100 uM) was used as a positive control.

## Competing interests

The authors declare that they have no competing interests.

## Authors' contributions

CG, JM and OT designed the study and drafted the manuscript. CG did the laboratory work and analyzed the data. EB and FE designed and supervised the prodigiosin purification procedures and critically revised the manuscript. All authors read and approved the final manuscript.
